# Evidence for height and immune function trade-offs among preadolescents in a high pathogen population

**DOI:** 10.1093/emph/eoaa017

**Published:** 2020-09-02

**Authors:** Angela R Garcia, Aaron D Blackwell, Benjamin C Trumble, Jonathan Stieglitz, Hillard Kaplan, Michael D Gurven

**Affiliations:** e1 Center for Evolution and Medicine, Arizona State University, 427 E Tyler Mall, Tempe, AZ 85281, USA; e2 School of Life Sciences, Arizona State University, 427 E Tyler Mall, Tempe, AZ 85281, USA; e3 Department of Anthropology, Washington State University, Pullman, WA 99163, USA; e4 School of Human Evolution and Social Change, Arizona State University, 900 Cady Mall, Tempe, AZ 85281, USA; e5 Université Toulouse 1 Capitole, espanade de l’Université 21, Allée de Brienne, Toulouse Cedex 06 31080, France; e6 Institute for Advanced Study in Toulouse, espanade de l’Université 21, Allée de Brienne, Toulouse Cedex 06 31080, France; e7 Economic Science Institute, Chapman University, One University Drive, Orange, CA 92866, USA; e8 Department of Anthropology, University of California, Santa Barbara, CA 93106, USA

**Keywords:** life history theory, developmental plasticity, ecological immunology, Tsimane

## Abstract

**Background:**

In an energy-limited environment, caloric investments in one characteristic should trade-off with investments in other characteristics. In high pathogen ecologies, biasing energy allocation towards immune function over growth would be predicted, given strong selective pressures against early-life mortality.

**Methodology:**

In the present study, we use flow cytometry to examine trade-offs between adaptive immune function (T cell subsets, B cells), innate immune function (natural killer cells), adaptive to innate ratio and height-for-age *z* scores (HAZ) among young children (*N* = 344; aged 2 months–8 years) in the Bolivian Amazon, using maternal BMI and child weight-for-height *z* scores (WHZ) as proxies for energetic status.

**Results:**

Markers of adaptive immune function negatively associate with child HAZ, a pattern most significant in preadolescents (3+ years). In children under three, maternal BMI appears to buffer immune and HAZ associations, while child energetic status (WHZ) moderates relationships in an unexpected direction: HAZ and immune associations are greater in preadolescents with higher WHZ. Children with low WHZ maintain similar levels of adaptive immune function, but are shorter compared to high WHZ peers.

**Conclusions:**

Reduced investment in growth in favor of immunity may be necessary for survival in high pathogen contexts, even under energetic constraints. Further, genetic and environmental factors are important considerations for understanding variation in height within this population. These findings prompt consideration of whether there may be a threshold of investment into adaptive immunity required for survival in high pathogen environments, and thus question the universal relevance of height as a marker of health.

**Lay Summary:**

Adaptive immune function is negatively associated with child height in this high pathogen environment. Further, low weight-for-height children are shorter but maintain similar immune levels. Findings question the relevance of height as a universal health marker, given that costs and benefits of height versus immunity may be calibrated to local ecology.

## 1. BACKGROUND AND OBJECTIVES

Energy available to organisms is limited and allocated to competing physiological demands in ways that optimize biological fitness. All else equal, energetic investment in one characteristic should trade-off with investment in other characteristics [1–3]. Natural selection can produce adaptations for managing trade-offs in species-typical ways, but it can also select for norms of reaction that manage allocations to fit local ecological contexts through developmental plasticity, allowing individuals to better approach the ‘optimal’ energy allocation strategy for a particular environment [4]. For example, given the strong selective pressure against infant mortality, early investment in immune function should be prioritized despite consequences for competing energetic requirements, like growth. This may be particularly salient in a high pathogen environment, where investment in the adaptive arm of the immune system, which confers long-lasting specific immunity, would be critical for surviving this early period [5].

Growth and immune trade-offs have been studied in humans and across vertebrate taxa [6]. In humans, numerous studies have shown that chronic immune activation [7, 8], and overall pathogen burden are associated with growth stunting [9, 10]. At young ages, while there may be early developmental origins of growth–immune associations, these relationships are often complex, and may be convoluted by changes occurring across multiple landscapes (e.g. maturational tempo, pathogen exposure, physical activity and diet) within a relatively narrow window of time. For example, in studies of infants in the Gambia [11] and Northern Kenya [12], researchers found that evidence of immune activation, as measured by (1) intestinal permeability and mucosal enteropathy and (2) immunoglobulin A (salivary), respectively, showed impaired growth, and that levels of immunoglobulins A, G and M rapidly increased with age [11]. Panter–Brick and colleagues similarly documented an age-related increase in IgG levels in a group of Nepali infants (*n* = 86), which varied by socioeconomic status, and was associated with lower weight-for-age [13]. However, the authors also found that mucosal damage, which was associated with impaired growth in height, was most severe among middle-class children, leading to the conclusion that nutritional constraints may mask or confound relationships between immune function and linear growth.

To date, many of the relevant studies have utilized either generalized markers of inflammation, or measures of immunoglobulins, and none that we are aware of include direct measurements of the leukocytes responsible for long-term adaptive immune function, including naïve and memory T cells and B cells. Numerous questions remain about how different aspects of immune function and activation relate to growth, whether these represent short- or long-term accommodations, and how factors like how nutritional environment, or energy availability, buffer or exacerbate these effects. Growth–immune trade-offs might represent short-term accommodations to temporary resource shortages, or longer-term phenotypic adjustments to expected future conditions [14]. In the short term, in addition to the direct costs of immune activation, anorexia associated with sickness behavior or gastrointestinal distress may cause sick children to eat less and absorb less nutrients [15], reducing energy available for growth. Particularly when intake is limited, fat stores may represent critical energy reserves upon which the body draws to fuel metabolic processes associated with growth and immune responses to infectious disease [16, 17]. Having larger energy intake or energy stores may allow for greater investment across multiple traits simultaneously. As a consequence, at the individual level there may be positive covariation among life history traits in individuals with ample resources [18]. In contrast to these short-term accommodations, long-term adjustments in expectation of future trade-offs may play out quite differently. For example, if short-term immune activation serves as a cue to future energy needs, we might expect individuals to shift energy into immune function and away from height, even in the absence of immediate energy shortages. We might also expect that short-term immune activation could lead to increases in energy storage, and therefore future fat accumulation [10].

One way to address the question of short- versus long-term accommodations and trade-offs is to examine multiple aspects of immunity that represent both short-term responses and long-term investments. Previous studies have found that different immune markers associate with growth on different timescales. For example, Urlacher *et al.* [10] found that a marker of acute inflammation associated with growth over one week, and was mediated by fat reserves, while immunoglobulin levels were associated with growth over months, and with overall height-for-age *z* scores (HAZ).

Here, we take a similar approach in examining both innate and adaptive markers. However, in contrast to past studies, we use flow cytometry to directly characterize leukocyte populations, the cells responsible for antibody production and cellular responses. This characterization includes both cells indicative of past exposures (non-naïve cells) and cells that represent immunological resources which have not yet been activated (naïve cells). In this article, we: (i) measure multiple immune markers in peripheral leukocytes (i.e. CD4+/CD8+, naïve and non-naïve and total T cells, B cells and natural killer (NK) cells) that characterize specific components of both adaptive and innate immune function and (ii) examine whether maternal or child energetic status, as measured through weight-for-height (WHZ) (child) and body-mass-index (mother), influence potential trade-offs between the child’s immune function and height (HAZ).

### 1.1 Measuring immune function in relation to growth–immune trade-offs

The human immune system is composed of innate and adaptive arms, which differ in their time-variant costs, time to reactivity and precision [19]. The arms of the immune system operate in an integrated way and in coordination with other organ systems (e.g. endocrine), with adaptive immune function in large part serving as an extension of, and directed by, the innate arm of the immune system [20, 21]. Innate immune functions are the body’s first-line non-specific defense mechanisms which are activated immediately upon encountering a foreign pathogen. Because they are generally applicable and immediately available, in many senses the costs of innate immunity may be seen as low, but innate responses also ultimately cause more collateral damage to surrounding cells and tissue, leading to faster rates of senescence [22–24]. By comparison, slower-acting adaptive immune responses require higher initial investment into the physiological mechanisms and systems that support them, including the production, selection and maintenance of pools of diverse B- and T-cell variants, many of which may never encounter a matching antigen and thus never be used [25, 26]. However, responses of the adaptive immune system are highly specialized, and usually cause little collateral damage. The adaptive immune system is also increasingly being recognized for its importance in immunological repair of cells and tissues [27]. The thymus, a major organ of the adaptive immune system, is the primary site of T cell development and maturation [28, 29]. That functional thymic volume peaks within the first year after birth, suggests this early period is a critical window for investment in adaptive immunity [28].

The innate immune response is highly sensitized, and can be hyper-variable across days due to even minor fluctuations in environmental stimuli [30]. This sensitization makes it hard to distinguish acute innate responses to short-term ecological stressors from longer-term calibration of the innate immune system to local ecological context. The relatively slower and less-generalized responses of the adaptive immune system suggest that, in cross-sectional studies, measures of adaptive immune function or the relative balance of adaptive to innate immune function may more reliably reflect the way the immune system is calibrated, and may thus provide a more accurate metric against which traits like growth may trade-off against.

**Box 1.** Components of the innate and adaptive immune system examined in this study

Immune arm	Main activity	Main target
Innate immunity		
NK cells	Kill missing-self or antigen-presenting cells	Viral; cell-mediated
Adaptive immunity		
T cells		
CD4+ (helper T)	Secrete cytokines and function to stimulate differentiation and proliferation of numerous immune cells	Aid in both cell-mediated and humoral processes
CD8+ (cytotoxic T)	Kill antigen-presenting cells	Tumor and virally-infected; cell-mediated
Naïve cells	Un-activated CD4+ or CD8+; have not yet encountered cognate antigen	Dependent on cell type
Non-naive cells	Activated CD4+ or CD8+; antigen- specific; long-lived	Dependent on cell type
B cells	Antibody production	Humoral

### 1.2 Hypothesis and predictions

Immuno-development early in life is in competition with growth, given that both require substantial energetic investment. We hypothesize that in high pathogen environments, where the cost of inadequate immune function is likely higher than smaller stature, we should see trade-offs between these life history features, and these trade-offs may be buffered by adequate resources. Specifically, we hypothesize that markers of adaptive immune function will trade-off against height, visible in this window of somatic and immunologic development, and that this trade-off will be moderated by energy availability. Data from the Tsimane, an energy-limited subsistence population in lowland Bolivia facing high pathogen loads [31], will be used to test the predictions that (P1) adaptive immune cell counts will be inversely associated with child HAZ. Furthermore, using the relative balance of investment into adaptive cellular immunity (i.e. B and T cell counts) to innate cellular immunity (i.e. NK cell counts) as a proxy for longer-term investment in costlier immune responses, we predict that (P2) a higher ratio of adaptive to innate immunity will associate with shorter stature. Finally, we predict that (P3) energetic status (proxied by child WHZ and maternal body mass index) will moderate associations between height and immune function, such that children with higher energetic status will be buffered and show a weaker trade-off (i.e. show weaker associations) between adaptive immune markers and height.

## 2. METHODOLOGY

### 2.1 Study population and data collection

#### 2.1.1 The Tsimane of Bolivia

The Tsimane are forager-horticulturalists (population ∼16 000) that live in the Beni Department of Bolivia, in the lowlands comprising the Amazon basin. Tsimane inhabit over 90 villages, most of which do not have access to electricity, running water or comprehensive waste management [32, 33]. Their diet is relatively lean, as they subsist mainly on plantains, rice, sweet manioc and corn from slash-and-burn horticulture, fish and wild game, occasionally supplemented by market goods such as refined sugar, salt and cooking oil [32, 34]. Despite evidence of child and adult stunting, wasting is uncommon; mean adult BMI is 23.6 for both sexes [31, 35]. From an early age, children are exposed to an array of pathogens, and parasitic infections among the Tsimane are common [36]. About half of Tsimane men and women have anemia, with children and adolescents showing the highest risk (56% for girls, 63% for boys) [37].

Given the high transmission rate of multiple pathogens in the Tsimane environment, investment in immune defenses is also high, with children exhibiting a concomitant early peak in humoral response (e.g. immunoglobulin-E, IgE) associated with helminthic infection compared to populations with lower transmission rates [36]. In general, Tsimane have higher levels of numerous immune components, including white blood cells (WBCs), erythrocyte sedimentation rate, B cells and NK cells than do Americans at all ages [31]. On average, 16% of WBCs are eosinophils, consistent with high levels of parasitic infection, as compared with a US reference range <5%, with approximately 85.9% of the population in eosinophilia by US standards [38]. Antibodies related to infection are also high among Tsimane: immunoglobulin-G (IgG) levels are about twice as high, and IgE is about 100 times higher than mean US levels [36].

#### 2.1.2 participants

Data for this study were collected as part of the Tsimane Health and Life History Project (THLHP), a longitudinal study of health and aging that began in 2002 [32]. The sample includes 344 children aged 2 months–8 years (55% female, median age 5 years) from 28 villages (see Table 1 for descriptive statistics). Participants were seen in the stationary THLHP clinic in San Borja (the closest market town to sampled villages), accompanied by a parent between mid-March and early July of 2011, whereupon participants were seen by THLHP physicians and given routine physical exams, which included patient history, symptom investigation, demographic and anthropometric information. A blood sample was taken as a part of this visit, a portion of which was used for assessing immune function.

**Table 1.** Descriptive statistics

Variables	Sample (*N* = 344)	
	Mean (SD)	Range
Sex (% female)	55	
Age (years)	4.71 (2.2)	0.15–8.07
Child's BMI (kg/m^2^)	16.18 (1.4)	11.19–23.11
Mother's height (cm)	150.62 (4.2)	140.7–165.0
Mother's BMI (kg/m^2^)	25.00 (3.5)	17.89–39.16
Respiratory infection (% infected)	47.8	
Parasite analyses (*n* = 246)		
Helminthic parasites (% infected)	56.6	
Protozoal parasites (% infected)	29.2	
Leukocytes (×10^3^ per µl)	11.98 (4.3)	4.30–33.60
T cell count (×10^3^ per µl)	3.07 (1.5)	0.74–11.07
Naïve T cell count (×10^3^ per µl)	2.27 (1.3)	0.38–8.51
CD4+ cell count (×10^3^ per µl)	1.85 (1.0)	0.40–6.17
CD8+ cell count (×10^3^ per µl)	1.22 (0.7)	0.10–5.61
B cell count (×10^3^ per µl)	1.28 (0.8)	0.22–6.91
NK cell count (×10^3^ per µl)	0.42 (0.3)	0.06–2.50

The study was approved by the Tsimane Government (Gran Consejo Tsimane) and village leaders. Study protocols were approved by the Tsimane Government and IRBs at the University of California, Santa Barbara (#12-496) and the University of New Mexico (#07-157). Adult participants provided informed consent for their own and their children’s participation. Children age six and older also provided verbal assent.

#### 2.1.3 Anthropometric and biomarker collection

Infant and child height (or length) were measured by trained Tsimane research assistants using a portable Seca infantometer or stadiometer (Hamburg, Germany). For the purposes of analysis, length and height were treated interchangeably. Weight was measured using a Tanita digital scale to the closest tenth of a kilogram. If infants were too small to stand on the scale, their weight was calculated by subtracting the weight of the mother from the combined weight of the mother holding her infant. Maternal height and weight measures were collected using the same instruments as used for the child, either at the same visit, or within 30 days of the child’s visit. Tsimane-specific *z*-scores for HAZ, child WHZ and maternal BMI-for-age (BMIZ) were calculated using the open-source R package *localGrowth* (https://github.com/adblackwell/localgrowth), which utilizes growth curves from [39]. These growth references better describe intra-population comparison by using locally-relevant growth trajectories [39]. Child WHZ and maternal BMIZ (in models for children under 3 years) were used as markers of energetic status, to model the effects of interactions between child energetic status and immune function on child HAZ. To determine the child’s age, exact birthdates were verified by the mother or pulled from longitudinal THLHP medical records.

##### 2.1.3.1 Biomarker collection

Blood was collected by a certified Bolivian biochemist into a heparin-coated vacutainer by venipuncture for children 2 years and older, and by capillary heel or finger prick for infants under 2 years of age [31]. Total WBC and hemoglobin were measured using a QBC Autoread Plus dry hematology system (QBC Diagnostics) directly after blood draw. Flow cytometry was performed in the THLHP clinic in San Borja on fresh heparinized blood within six hours of the blood draw in order to quantify lymphocyte subsets into helper T cell (CD4+CD8−), cytotoxic T cells (CD8+CD4−), NK cells (CD56+CD8−CD4−) and B cells (CD19+) using an Accuri C6 Flow Cytometer (BC Accuri Cytometers). T cells were further classified as naïve (CD45RA+) or non-naïve (CD45RA−). Absolute counts were calculated by multiplying the relative percentages determined by flow cytometer by the total lymphocyte count obtained from the QBC Autoread Plus. For full description of gating and antibodies used [31].

##### 2.1.3.2 Parasite analyses

Fecal samples were analyzed for the presence of helminth eggs and larvae by direct identification on wet mounts or by a modified Percoll method [9, 40]. Parasite infection was recorded as presence or absence of each species, and is based on a single fecal sample, collected and analyzed at the health clinic within one day of blood collection.

### 2.2 Statistical analysis

Mixed effects models fit with restricted (residual) maximum likelihood (REML), from the R package *lme4,* were used to evaluate relationships between markers of immune function and child HAZ. Maternal ID was included as a random effect, as a means to parse contribution of potential genetic clustering or clustering due to immediate environment (e.g. shared household) from individual-level factors that contribute to overall variation in HAZ [41]. Effects of community-level clustering were also considered, and initial models included community ID as a random effect. However, since the inclusion of community ID did not substantially improve fit for any model, it was not included in final analyses. Height *z*-scores for mothers are also included in models, for additional adjustment for potential heritable variation in height. To adjust for variation in immune function based on current infection and associated immune activation, we include covariates for respiratory illness and residual WBC counts. Additional models were ran using the subset of the sample for whom fecal parasite data were available (72% of sample), which include covariates for current protozoal and helminthic infections assessed from fecal samples. For each model, residual WBC count is WBC count minus the portion of cells that were being used as the main independent variable of interest (e.g. in a model testing the association between total T cell count and height, residual WBC = total WBC − T cell count). This adjustment is necessary to minimize collinearity. To measure relative investment in adaptive relative to innate immunity, we calculate a ratio of adaptive (B and T) cells to innate (NK) cells. Due to skewed distributions, all immune cell counts are log-transformed and standardized (*z*-scored) before inclusion in regression models. All analyses were performed using R statistical software version 3.4.2 [43]. All models adjust for age and sex. All results are reported as standardized betas unless otherwise noted. Both marginal and conditional R-squared values are included in tables for linear mixed effects model results. While marginal R-squared reflects the variance explained only by model fixed effects, the conditional R-squared is interpreted to reflect variance explained by both fixed and random effects (see Ref. [42] for discussion of limitations on the use of these R-squared estimates in mixed effects models).

## 3. RESULTS

Children in this study ranged from 2 months to 8 years of age (mean: 4.7 years, SD: 2.2) and 55% were female. Overall, 72% of participants were screened for parasites. Of this sample, 57% of children were infected with at least one species of parasite, with 25.3% having coinfections with more than one species. The most common infections were helminths, with hookworm (*Ancylostoma duodenale* or *Necator americanus*, prevalence 50.6%) being most common, followed by roundworm (*Ascaris lumbricoides*, 16.1%), threadworm (*Strongyloides stercoralis*, 7.7%) and whipworm (*Trichuris* sp., 2.3%). Protozoan infections were also common, including *Giardia lamblia* (24.1%) and *Entamoeba histolytica* (8.4%); further, 47.8% of children were diagnosed as having a current respiratory condition. Using WHO criteria for age-specific anemia cutoffs, 45.7% of children also had at least mild anemia (<11 mg/dl of hemoglobin in children under age 5, and <11.5 mg/dl for children aged 5–11 years).

### 3.1 Age-related changes in lymphocyte subsets

**Figure 1.**
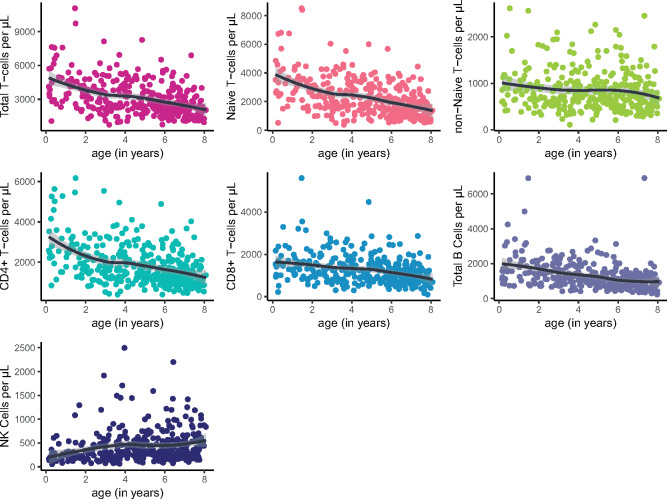
(**a**–**g**) Total T cells, including naïve and non-naïve, CD4 and CD8 subsets and B cells, significantly decline with age, while NK cells do not. The trendlines are loess fit (span = 0.75)

We first examined overall differences in lymphocyte subsets by age, adjusting for sex and current infections, fit with smoothed loess curves based on generalized linear models (Fig. 1). Total T cells (*β* = −294.89 cells/μl of blood/year, *P* < 0.001) and all subsets (naïve: *β* = −264.94, *P* < 0.001; non-naïve: *β* = −29.96, *P* < 0.01; CD4^+^: *β* = −191.73, *P* < 0.001; CD8^+^: *β* = −103.19, *P* < 0.001) and B cells (*β* = −145.85, *P* < 0.001) significantly decline with age. Conversely, NK cells increase with age (*β*  =  30.85, *P* < 0.001).

### 3.2 Associations between adaptive immune function and child height

**Figure 2.**
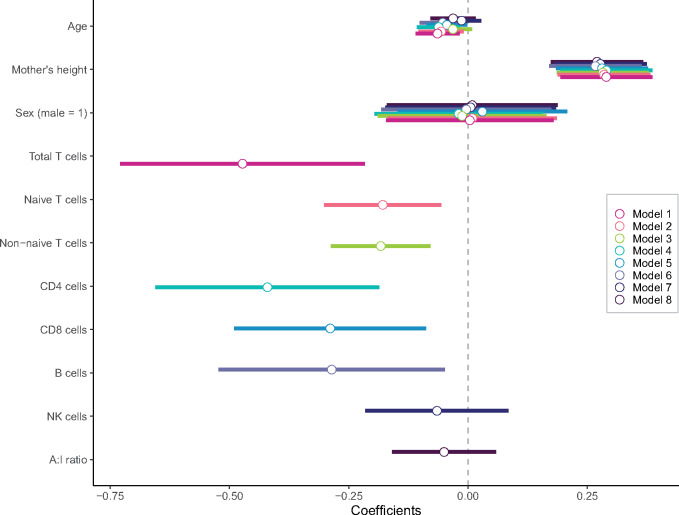
Trade-offs between child height and adaptive immunity. This figure compares parameter estimates of associations between measures of adaptive immunity and child height across all adaptive immune models from Table 2. All immune markers were logged and standardized before analysis; A:I ratio is the ratio of adaptive to innate immune cells, logged and standardized for analysis. Analyses include controls for age, mother’s height, sex and WBC (logged and *z*-scored). *P < 0.05; **P < 0.01; ***P < 0.001

Mixed effects linear regression models are used to evaluate relationships between child HAZ and markers of immune function, with a random effect for mother’s ID (Fig. 2 and Table 2). HAZ is consistently negatively associated with adaptive immune cell counts after adjusting for age, sex, mother’s height, residual WBC and current respiratory illness. Higher HAZ children have lower overall T cells (*β* = −0.37, *P* = 0.001), including both naïve (*β* = −0.15, *P* = 0.009) and non-naïve (*β* = −0.16, *P* = 0.001) T cell counts, CD4+ (*β* = −0.33, *P* = 0.002) and CD8+ (*β* = −0.27, *P* = 0.006) subsets (Fig. 2: *Models 1–5*) and lower B cell counts (*β* = −0.25, *P* = 0.03; *Model 6*). There is no association between innate (i.e. NK) cell counts and height (*β* = −0.05, *P* = 0.48; *Model 7*). Further, a higher ratio of adaptive to innate immune cells is not associated with child height (*β* = −0.05, *P* = 0.35) (Fig. 2: *Model 8*). Including maternal ID significantly increased model fit across all models (Table 2), suggesting that genetic and/or shared environmental factors influence variation in child HAZ.

**Table 2.** Associations between immune function and child HAZ

	Dependent variable: height (*z*-scored)							
	Total T	Naïve	Non-naïve	CD4+	CD8+	B cells	NK cells	A:I ratio
Cell counts^a^	−0.372**	−0.149**	−0.163**	−0.334**	−0.269**	−0.254*	−0.053	−0.052
	(0.115)	(0.057)	(0.050)	(0.107)	(0.096)	(0.115)	(0.076)	(0.056)
Age (in years)	−0.058*	−0.053*	−0.032	−0.056*	−0.043	−0.045	−0.008	−0.026
	(0.025)	(0.026)	(0.022)	(0.025)	(0.024)	(0.026)	(0.022)	(0.025)
Sex (male = 1)	0.038	0.043	0.016	0.018	0.060	0.024	0.030	0.036
	(0.093)	(0.094)	(0.093)	(0.093)	(0.094)	(0.094)	(0.094)	(0.094)
Mother's height^b^	0.294***	0.291***	0.287***	0.293***	0.287***	0.271***	0.279***	0.273***
	(0.051)	(0.051)	(0.051)	(0.051)	(0.051)	(0.051)	(0.051)	(0.051)
Residual WBC^c^	0.218	0.181	0.207	0.214	0.163	0.169	0.017	−0.009
	(0.124)	(0.123)	(0.123)	(0.124)	(0.121)	(0.148)	(0.137)	(0.134)
Respiratory^d^	−0.125	−0.135	−0.099	−0.135	−0.113	−0.101	−0.112	−0.112
	(0.092)	(0.094)	(0.094)	(0.092)	(0.092)	(0.093)	(0.093)	(0.093)
Constant	−1.572	−1.251	−1.588	−1.535	−1.156	−1.227	0.004	0.267
	(1.076)	(1.069)	(1.078)	(1.077)	(1.054)	(1.321)	(1.279)	(0.342)
Observations	344	343	343	344	344	344	344	344
Log Likelihood	−442.31	−443.89	−442.22	−442.68	−443.76	−445.12	−447.70	−447.79
R-squared (m)^e^	0.13	0.12	0.13	0.12	0.12	0.11	0.10	0.10
R-squared (c)^f^	0.29	0.28	0.29	0.29	0.28	0.27	0.26	0.26
Akaike Inf. Crit.	902.62	905.77	902.44	903.37	905.53	908.24	913.40	913.58
ANOVA table for random effect: Mother’s ID								
Log likelihood	−445.44	−447.05	−445.48	−445.71	−446.90	−448.10	−450.8	−450.74
Akaike Inf. Crit.	906.88	910.10	906.95	907.42	909.80	912.21	917.6	917.48
LRT^g^	6.26*	6.32*	6.51*	6.05*	6.27*	5.97*	6.20*	5.91*

*Notes:* REML regressions modeling associations between immune cell count and child HAZ.

a All immune measures are logged and standardized,

b maternal height is *z*-scored,

c residual WBC is fraction of WBC not included in cell count (e.g. for Mod 1, WBC = total WBC – T cell count), *z*-scored,

d clinician diagnosed respiratory infection (yes = 1),

e marginal *r*-squared represents the variance explained by the fixed effects,

f conditional *r*-squared represents the variance explained by both the fixed and random effects,

g likelihood ratio test statistic.

* *P* < 0.05;

** *P* < 0.01;

*** *P* < 0.001.

### 3.3 Height and immune associations by age group

Given children younger than 3 years are likely still supplemented both immunologically and energetically by their mothers through breastfeeding, the next set of analyses examines associations between HAZ and immune cell counts including an interaction with age group. For these analyses, children are split into two groups: infants (children 2 months to 3 years; *n* = 82) and preadolescents (children 3–8 years; *n* = 261). There are no significant associations between HAZ and immune cells across cell types in infants (Table 3). In contrast, for preadolescents, HAZ and total T cells (*β* = −0.49, *P* < 0.001), both naïve (*β* = −0.20, *P* = 0.002) and non-naïve (*β* = −0.20, *P* < 0.001) T cell counts, CD4+ (*β* = −0.47, *P* < 0.001) and CD8+ (*β* = −0.32, *P* = 0.004) T cell subsets, and B cells (*β* = −0.34, *P* = 0.006), but not NK cells (*β* = −0.03, *P* = 0.73) or A:I ratio (*β* = −0.10, *P* = 0.10) (Table 3). Because children traverse through numerous developmental phases even between 3 and 8 years of age, we ran additional models which included a finer-grained age-category variable, splitting children into approximately equally weighted age groups (2 months to 3 years, *n* = 82; 3–5 years, *n* = 91; 5–6.5 years, *n* = 80; 6.5–8 years, *n* = 90), and found that these associations were strongest among the two middle groups (3–5 and 5–6.5 years) (Supplementary Table S1).

**Table 3.** Associations between immune function and child HAZ by age group

	Dependent variable: height (*z*-scored)							
	Total *T*	Naïve	Non-naïve	CD4+	CD8+	B cells	NK cells	A:I ratio
Age (in years)	−0.048	−0.041	−0.027	−0.046	−0.038	−0.032	−0.005	−0.009
	(0.025)	(0.026)	(0.022)	(0.025)	(0.024)	(0.026)	(0.023)	(0.027)
Sex (male = 1)	0.044	0.048	0.022	0.018	0.067	0.027	0.028	0.033
	(0.092)	(0.093)	(0.093)	(0.092)	(0.094)	(0.093)	(0.094)	(0.094)
Mother’s height	0.291***	0.287***	0.286***	0.288***	0.285***	0.273***	0.276***	0.264***
	(0.051)	(0.052)	(0.051)	(0.051)	(0.051)	(0.051)	(0.052)	(0.051)
Residual WBC	0.230	0.191	0.201	0.237	0.159	0.149	0.024	0.001
	(0.123)	(0.123)	(0.123)	(0.124)	(0.121)	(0.148)	(0.138)	(0.134)
Respiratory (yes = 1)	−0.141	−0.151	−0.109	−0.157	−0.119	−0.110	−0.110	−0.115
	(0.092)	(0.094)	(0.094)	(0.092)	(0.093)	(0.092)	(0.093)	(0.093)
Cell count: 3 years and over	−0.494***	−0.199**	−0.202***	−0.470***	−0.323**	−0.339**	−0.030	−0.103
	(0.129)	(0.063)	(0.056)	(0.120)	(0.107)	(0.123)	(0.085)	(0.062)
Cell count: under 3 years	−0.025	0.003	−0.038	0.045	−0.087	0.055	−0.134	0.109
	(0.203)	(0.102)	(0.095)	(0.189)	(0.183)	(0.200)	(0.149)	(0.103)
Constant	−1.749	−1.423	−1.567	−1.818	−1.159	−1.134	−0.082	0.125
	(1.073)	(1.068)	(1.076)	(1.075)	(1.053)	(1.317)	(1.288)	(0.349)
Observations	344	343	343	344	344	344	344	344
Log Likelihood	−440.75	−443.56	−442.34	−440.43	−443.77	−444.00	−448.38	−447.32
R-squared (m)	0.14	0.12	0.13	0.14	0.12	0.12	0.10	0.11
R-squared (c)	0.31	0.30	0.30	0.31	0.29	0.28	0.26	0.27
Akaike Inf. Crit.	901.49	907.12	904.68	900.85	907.54	908.00	916.76	914.64

*Notes:* REML regressions modeling interaction effects of immune cell count and age on child HAZ. Results are reported as beta coefficient (standard error). Cell counts are logged and *z*-scored; Mother’s height is *z*-scored; Residual WBC is fraction of WBC not included in cell count (e.g. for Mod 1, WBC = total WBC – T cell count), *z*-scored. ‘Respiratory’ is a binary variable (yes = 1) representing current respiratory illness, as diagnosed by a physician at time of visit. R-squared (m) is the marginal r-squared and represents the variance explained by the fixed effects; R-squared is the conditional r-squared and represents the variance explained by both the fixed and random effects.

* *P* < 0.05;

** *P* < 0.01;

*** *P* < 0.001.

### 3.4 maternal and child energetic status as a moderator of immune-height trade-offs

**Figure 3.**
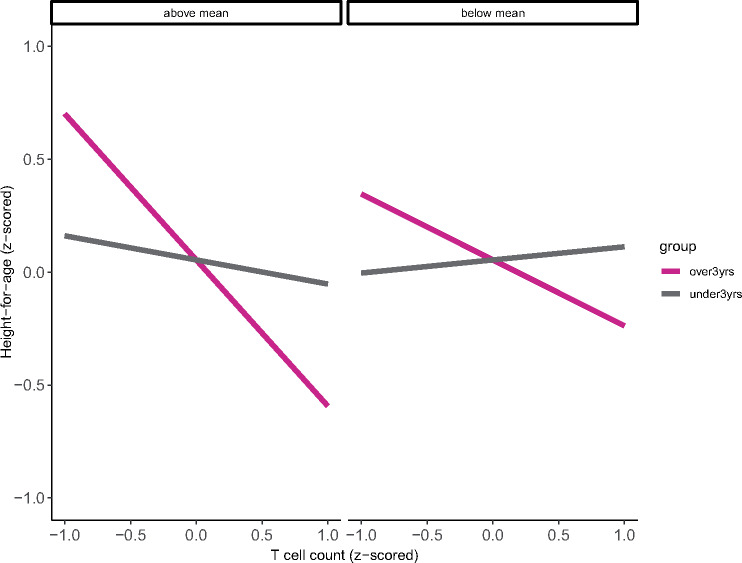
Graphical display of results from Table 4, *model 1.* For children 3 years and older, there is a negative interactive effect of child WHZ on the association between total T cell count and height. There appears to be a height-immune trade-off only in children that are at or above the mean for WHZ. Note: child height and WHZ are *z*-scored, and total T cell count is logged and *z*-scored for analyses. Model controls for age, sex, mother’s height and current immune activation, and a random effect for maternal ID

To test the prediction that having adequate energetic reserves may buffer a trade-off between immune function and growth in the child, we examine the effects of a three-way interaction between each immune marker, child or maternal energetic status and age group on child HAZ. In children under 3 years old, maternal energetic status (maternal BMIZ) moderates the relationship between total T (*β*  =  0.31, *P* = 0.08) and CD4+ T cell subsets (*β*  =  0.33, *P* = 0.05), and the A:I ratio (*β*  =  0.19, *P* = 0.04) and height, but does not show any influence on immune cell counts and height relationships in preadolescents (Supplementary Table S2). In other words, for infants whose mothers have above the mean BMI, there is a positive association between cell count and HAZ, whereas for older children (preadolescents), there remains a negative association between cell count and HAZ, regardless of maternal BMI (Supplementary Fig. S1). Alternately, models with child energetic status, measured by the child’s WHZ, show a different pattern. For preadolescents (but not infants), there are interaction effects whereby WHZ status influences associations between height and total T cell (*β* = −0.42, *P* < 0.005), naïve (*β* = −0.23, *P* = 0.001), CD4+ (β = −0.41, *P* = 0.002) and CD8+ cell counts (*β* = −0.25, *P* = 0.06) (Table 4). Contrary to our predictions, the parameter estimates for these interaction terms are negative: children with lower (i.e. below mean) WHZ show a reduced association between adaptive immune function and HAZ compared with high (i.e. above mean) WHZ children (Fig. 3: total T cells). In fully adjusted models, high WHZ children are taller for their age compared to low WHZ children (*β*  =  0.24, *P* = 0.01; Supplementary Fig. 2), but do not differ across measures of adaptive immune function (total T cells: *β*  =  0.03, *P* = 0.53; naïve: *β*  =  0.10, *P* = 0.28; non-naïve: *β* = −0.03, *P* = 0.80; CD4: *β*  =  0.03, *P* = 0.58; CD8: *β*  =  0.01, *P* = 0.86; or B cells: *β* = −0.01, *P* = 0.88). Child WHZ does not directly predict differences in HAZ across any model (Supplementary Table S3), and in fact lowers the model fit (measured by R-squared). Again, in all models, mother’s height and maternal ID have significant effects on child’s HAZ.

**Table 4.** Associations between immune function, WHZ, and child HAZ

	Dependent variable: height (*z*-scored)							
	Total *T*	Naïve	Non-naïve	CD4+	CD8+	B cells	NK cells	A:I ratio
Age (in years)	−0.013	−0.012	−0.016	−0.015	−0.013	−0.012	−0.014	−0.014
	(0.022)	(0.021)	(0.022)	(0.021)	(0.022)	(0.021)	(0.021)	(0.021)
Sex (male = 1)	0.018	0.019	0.035	0.016	0.031	0.032	0.031	0.024
	(0.094)	(0.093)	(0.095)	(0.093)	(0.094)	(0.095)	(0.094)	(0.094)
Mother’s height	0.289***	0.291***	0.281***	0.286***	0.286***	0.289***	0.276***	0.281***
	(0.051)	(0.051)	(0.052)	(0.051)	(0.051)	(0.052)	(0.051)	(0.051)
Residual WBC	0.063	0.062	0.075	0.075	0.053	0.013	−0.004	−0.027
	(0.116)	(0.116)	(0.118)	(0.116)	(0.117)	(0.130)	(0.133)	(0.134)
Respiratory	−0.113	−0.112	−0.103	−0.116	−0.109	−0.103	−0.112	−0.099
	(0.093)	(0.094)	(0.096)	(0.093)	(0.093)	(0.093)	(0.094)	(0.094)
Cell count: 3 years +: child WHZ	−0.419**	−0.236**	−0.026	−0.406**	−0.255*	−0.181	0.042	−0.112
	(0.149)	(0.072)	(0.060)	(0.133)	(0.134)	(0.119)	(0.091)	(0.065)
Cell count: <3 years: child WHZ	−0.242	−0.114	−0.127	−0.245	−0.186	−0.204	−0.065	−0.052
	(0.168)	(0.081)	(0.091)	(0.162)	(0.158)	(0.153)	(0.137)	(0.084)
Constant	−0.360	−0.354	−0.484	−0.460	−0.291	0.066	0.226	0.252
	(1.025)	(1.023)	(1.037)	(1.022)	(1.034)	(1.183)	(1.232)	(0.340)
Observations	344	343	343	344	344	344	344	344
Log Likelihood	−443.15	−442.46	−447.69	−442.50	−445.73	−446.42	−448.61	−447.96
R-squared (m)^e^	0.12	0.13	0.10	0.13	0.11	0.11	0.10	0.11
R-squared (c)^f^	0.29	0.30	0.27	0.30	0.28	0.27	0.26	0.27
Akaike Inf. Crit.	906.31	904.93	915.37	905.00	911.47	912.85	917.23	915.91

*Notes:* REML regressions modeling three-way- interaction effects of immune cell count, age, and child energetic status (proxied by WHZ) on child HAZ. Results are reported as beta coefficient (standard error). Cell counts are logged and *z*-scored; Mother’s height is *z*-scored; Residual WBC is fraction of WBC not included in cell count (e.g. for Mod 1, WBC = total WBC – T cell count), *z*-scored. ‘Respiratory’ is a binary variable (yes = 1) representing current respiratory illness, as diagnosed by a physician at time of visit. R-squared (m) is the marginal r-squared and represents the variance explained by the fixed effects; R-squared is the conditional r-squared and represents the variance explained by both the fixed and random effects.

* *P* < 0.10;

** *P* < 0.05;

*** *P* < 0.01;

*** *P* < 0.001.

### 3.5 Influence of current parasitic infection on HAZ and immune associations

Though in general, Tsimane children face relatively high exposure to parasites, variation in current infection may influence physiological aspects of any particular child, including immune cell repertoire and energetic status. Further, a child’s exposure may also vary based on her/his age and stage of development. As such, we conduct additional analyses on the subset of children in this sample for whom data on current infection was available (*n* = 246, 72% of total sample). Models include dichotomous covariates for (presence/absence of) helminthic parasites (hookworm, roundworm, threadworm, whipworm) and protozoal parasites (*G. lamblia* and *E. histolytica*). In these models, adjusting for helminthic and protozoal infections strengthens inverse associations between immune cells and HAZ across all cell types, except for the adaptive:innate ratio (Supplementary Table S4). When binned by age group, adjusting for parasitic infections increases the statistical significance of negative associations between all adaptive immune cells and HAZ in preadolescence, and though it increases the effect size across most cell types in infants, none reach statistical significance (Supplementary Table S5). Based on analysis of covariance models which adjust for age effects, neither helminthic nor protozoal infection directly predicted differences in immune cells or WHZ (Supplementary Table S6). However, infants are significantly less likely to be infected by helminths, compared to preadolescents (*B* = −1.44, *P* < 0.001), but show no difference in protozoal infection (*B* = 0.32, *P* = 0.31).

## 4. DISCUSSION

The aim of this study is to further our understanding of life history trade-offs between growth and immune function in early childhood, and the potential role of energetic status (maternal and child) in influencing these relationships. This study incorporates a diverse array of immune markers of specific immune function, including lymphocyte subtypes (naïve, non-naïve and total T cells, and CD4+ and CD8+ subtypes and B cells), and the ratio of adaptive to innate (NK cells) immune cell counts as a measure of longer-term allocations to immunity.

We find partial support for the hypothesis that a high pathogen environment should favor early investment in adaptive immune function, and that such heavy investment may trade-off against somatic growth. In support of our first prediction, higher counts of adaptive immune cells are associated with shorter stature (measured by HAZ) across all T cell populations and B cells. Associations are further strengthened when adjusting for current parasitic infection. These findings complement experimental work from animal literature as well as research among human populations living in high pathogen environments. For example, magpies that were given methionine, a sulfur amino acid that enhances T cell immune response, exhibited less growth over the course of treatment than controls [44]. In humans, other research among the Tsimane, and the Shuar of Ecuador, both of which face relatively high parasite loads, reports consistent growth and immune function trade-offs. In both populations, inverse associations are found between markers of adaptive and innate immune function with both cross-sectional HAZ and longitudinal growth. Higher levels of immunoglobulin E, a measure of adaptive humoral immune and indicator of past exposure to parasites, was associated with lower HAZ scores [9], while higher levels of C-reactive protein were associated with reduced longitudinal growth [17]. Further, among the Shuar, immunoglobulin G and C-reactive protein were also inversely associated with longitudinal growth [10].

That both naïve and non-naïve T cells similarly negatively associate with HAZ in our sample suggests that growth–immune trade-offs are not strictly due to past exposures. Naïve T cells have not yet encountered antigens, and reduced cell counts might represent past exposures, if exposure converts naïve to non-naïve cells. However, if this were the case, we would expect a positive association between T cells and height, assuming infection itself is costly. In contrast, higher naïve T cell counts are likely to represent investment in immune defenses for threats that have not yet been encountered. The fact that both cell types associate negatively with height, even in models that adjust for current parasitic infection, suggests that trade-offs with height are likely due to overall investment in cellular immunity and are not necessarily dependent on the causes of that investment (i.e. responsive vs. predictive). These associations suggest that it is the immune investment itself may trade-off with height, rather than direct costs of past infections.

Though in the full sample there are negative associations between adaptive immune markers and HAZ, when children are split into age-groups: infants (< 3 years old) and preadolescents (3-8 years old), cell counts and HAZ associations we find that are not statistically significant in the younger age group. The lack of an association between HAZ and immune function in infants may be due to a few statistical or biological facets. Statistically, because coefficients were consistently negative (as in the older group) after adjusting for current immune activation, the lower sample size and higher error in this group may have obscured any statistical significance in associations from emerging. Biologically, it may be that infants maintain adequate buffering by maternal energetic and immunologic resources, transmitted via breastfeeding, through this time period. In accordance with this idea, we do find a positive interaction between mother’s energetic status and associations between immune cell counts and HAZ among infants whereby children with higher-BMI moms show a positive association between height and immune cell counts. There is a rich literature showing growth faltering after weaning, which may be a consequence of increased exposure to contaminants and other environmental pathogens [45–47]. In fact, we do see that preadolescents are more likely to have helminthic parasites compared to infants, and, that protozoal infection is positively associated with greater WHZ, which in infants may be evidence of earlier weaning. Though we do not have systematic quantitative data on breastfeeding status and intensity for the present sample, a different Tsimane study found that children breastfeed until between 30-36 months of age, in concert with the introduction of complementary foods [48].

However, another possibility is that the lack of associations could be due to heterogeneity in maturational pace among individuals in this age group. For example, children who are maturationally delayed overall, would be smaller and likely have higher naïve immune cell counts. Growth is not continuous, and it is therefore difficult to confidently assess the impact of current immune profiles on growth using cross-sectional measures. It is likely that there are coordinated developmental changes across these various systems (height, weight, immune status) that vary by both chronological and maturational age which obscure our ability to tease apart trade-offs from shifts in coordination. In these data, we found that in fact the largest effect sizes for associations between immune cells and HAZ was among those aged 3–6.5 years old. However, given the relatively small sample sizes in each age category, a deeper evaluation of coordinated shifts among physiological systems across age was out of the scope of this article. Additional research with larger sample sizes are needed to fully address this issue.

Regarding our last prediction, we found that among preadolescents, child energetic status (WHZ) did moderate associations between HAZ and immune cell counts across all T-cell subtypes, and that inverse associations between all adaptive cell types and height are strengthened after accounting for interactive effects of WHZ and immune markers. However, contrary to our prediction, the direction of the interaction term was negative. In graphing these interactions, we found that negative height and immune associations are more significant in children with higher nutritional status. At the proximal level, this is likely explained by the fact that lower WHZ children are, on average, shorter for their age than high WHZ children, but do not show differences across measures of adaptive immune function.

That children with reduced nutritional status are shorter but have similar adaptive immune profiles may allude to a ‘lower threshold’ for investment in adaptive immune function that is critical for survival in high pathogen environments which must be maintained in the face of energetic constraints. Thus, additional resources might be invested in height, but not at a cost to meeting the baseline for adequate adaptive immune function. Though we cannot conclude this from the current data, this speculation fits well within the immunology literature that posits an optimality in bounded, or ‘intermediate’, variation in components of pathogen recognition systems like MHC complexes [49]. Similarly in evolutionary ecology, a number of studies have documented population-level variation in immune-related traits driven by ecological pressures like pathogen diversity, which may vary spatially [50] and seasonally [51]; for a review see [52]. Studies among the Tsimane have also consistently found upregulation in most components of immune function relative to ‘standard cutoffs’ in the U.S., and directly compared to populations under less pathogen exposure, including from the U.S. and Ecuador ([36]; [53]). It is important to note that this is not the same idea as ‘phenotypic correlation’—which suggests that a positive correlation between two traits can occur when individuals with plentiful resources can allocate excess quantities to both traits [54]. In fact, this idea is complementary by suggesting that there may be a relatively stable lower limit to the level of investment in immune function that must be met to ensure survival, which should be dependent on ecological context and likely differ across populations.

However, an alternate interpretation is that pathogenically-stressed or ill children may eat less or absorb less nutrients, which may have effects on growth and immune function. In such cases, children may show growth faltering [55, 56], and greater immune responsivity, yet have overall attenuated immune function [57]. While this could plausibly explain some variation in height and immune function, the findings reported here hold after the inclusion of numerous covariates to adjust for differences in current pathogen exposure and immune activation, and thus we argue that adding an evolutionary perspective to this question is important for expanding how we think about variation in life history traits and health.

Finally, we find significant fixed and random effects of variables that capture genetic and shared environmental factors (e.g. maternal height and ID, and current parasitic infection). Across most models, conditional R-squared, which represents the variance explained by both fixed effects and a random effect for maternal ID captures roughly 10% more variance in HAZ than fixed effects alone. Further, models that adjust for maternal factors and parasitic infection capture over 30% of variation in HAZ scores across all models. These findings point to a confluence of factors that interact and influence height differences between children, and highlight the fact that growth and immune trade-offs, while possible, are nowhere near zero-sum.

### Limitations

Though we believe this study is novel in its application of ecological immunology to inform our understanding of trade-offs between growth and immunity, we are cautious about making strong inferences. We recognize that a cross-sectional study design limits our ability to establish causality, and tease apart short-term trade-offs versus longer-term phenotypic adjustments, such as a predictive adaptive response [58]. Further, there may be issues with using WHZ as a proxy for energetic status, given that these measures do not adequately reflect variation in fat-free body mass from body fat. Higher activity level (and thus muscle mass and WHZ) may also reduce energy available for growth, in addition to the costs of immune function. Though this is not necessarily the case, this possibility cannot be ruled out. Along the same vein, both growth and energy reserves may not be stable over time, particularly during early windows of development. However, the child population-specific growth curves for the Tsimane have relatively constant WHZ velocities until around the age of 10 years [39].

## 5. CONCLUSION AND IMPLICATIONS

Tracking trade-offs between growth and immune function is enormously complex, particularly due to the changing relationships between distribution of body fat and mass relative to height, and shifts in allocation between life history traits, that occur during this period of active growth and development as growing bodies attempt to calibrate optimally to their projected environment. In this study, we find that among children in a high-pathogen environment, greater investment in aspects of adaptive immune function is negatively associated with HAZ. Child energetic status appears to moderate this association, and the strongest negative associations are found in older children with higher nutritional status. Children with lower WHZ are shorter but retain high investment in adaptive immunity, indicating the biological significance of maintaining a robust adaptive immune system even under energetic constraints. Reduced investment in growth in favor of immunity may be necessary for survival in high pathogen contexts. Further, genetic and (shared) environmental factors are important considerations for understanding variation in height within this population. The implications of these findings may be important for prompting a reconsideration of how height is used as a marker of health. Given the high plasticity and variable heritability in height, in conjunction with the cost and utility of immune function across environments, stature may not be a universally equitable metric for predicting health.

## SUPPLEMENTARY DATA

Supplementary data is available at *EMPH* online.

## Supplementary Material

eoaa017_Supplementary_Data
