# Supplementary material for: Evidence for height and immune function trade-offs among preadolescents in a high pathogen population
Source: Evol Med Public Health. 2020 Sep 2;2020(1):86–99. doi: 10.1093/emph/eoaa017 (PMC7502263; doi:10.1093/emph/eoaa017)
Supplement: eoaa017_Supplementary_Data [file eoaa017_supplementary_data.docx]

| **Supplemental Table 1.** | | | | | | | | | | | |
| --- | --- | --- | --- | --- | --- | --- | --- | --- | --- | --- | --- |
|  | Dependent variable: Height (z-scored) | | | | | | | | | | |
|  | | Total T | Naïve | Non-naïve | CD4+ | CD8+ | B cells | NK cells | A:I ratio | |  |
| Age (in years) | | -0.044 | -0.041 | -0.024 | -0.043 | -0.035 | -0.029 | -0.009 | -0.009 | |  |
|  | | (0.026) | (0.027) | (0.022) | (0.026) | (0.024) | (0.027) | (0.023) | (0.027) | |  |
| Sex (male = 1) | | 0.046 | 0.049 | 0.029 | 0.020 | 0.068 | 0.029 | 0.027 | 0.025 | |  |
|  | | (0.092) | (0.093) | (0.093) | (0.092) | (0.094) | (0.093) | (0.095) | (0.094) | |  |
| Mother’s height | | 0.294^***^ | 0.288^***^ | 0.291^***^ | 0.291^***^ | 0.289^***^ | 0.279^***^ | 0.271^***^ | 0.257^***^ | |  |
|  | | (0.051) | (0.052) | (0.051) | (0.051) | (0.052) | (0.051) | (0.052) | (0.052) | |  |
| Residual WBC | | 0.240 | 0.193 | 0.210 | 0.244 | 0.171 | 0.170 | 0.026 | -0.002 | |  |
|  | | (0.124) | (0.124) | (0.123) | (0.124) | (0.122) | (0.149) | (0.138) | (0.134) | |  |
| Respiratory (yes = 1) | | -0.147 | -0.152 | -0.109 | -0.164 | -0.120 | -0.110 | -0.118 | -0.122 | |  |
|  | | (0.093) | (0.096) | (0.094) | (0.093) | (0.093) | (0.093) | (0.094) | (0.094) | |  |
| Cell Count : [0.14,3yrs] | | -0.019 | 0.003 | -0.037 | 0.051 | -0.089 | 0.049 | -0.131 | 0.106 | |  |
|  | | (0.203) | (0.102) | (0.095) | (0.190) | (0.183) | (0.201) | (0.149) | (0.104) | |  |
| Cell Count : (3,5yrs] | | -0.595^**^ | -0.210^*^ | -0.269^***^ | -0.519^**^ | -0.423^*^ | -0.493^*^ | -0.007 | -0.163 | |  |
|  | | (0.200) | (0.095) | (0.082) | (0.182) | (0.179) | (0.207) | (0.130) | (0.100) | |  |
| Cell Count : (5,6.5yrs] | | -0.506^*^ | -0.198 | -0.221^*^ | -0.512^*^ | -0.317 | -0.360 | -0.146 | -0.008 | |  |
|  | | (0.220) | (0.108) | (0.103) | (0.205) | (0.201) | (0.213) | (0.140) | (0.111) | |  |
| Cell Count : (6.5,8.08yrs] | | -0.372 | -0.186 | -0.081 | -0.380^*^ | -0.243 | -0.220 | 0.092 | -0.118 | |  |
|  | | (0.209) | (0.105) | (0.101) | (0.189) | (0.165) | (0.174) | (0.158) | (0.100) | |  |
| Constant | | -1.852 | -1.441 | -1.666 | -1.882 | -1.273 | -1.332 | -0.079 | 0.147 | |  |
|  | | (1.083) | (1.078) | (1.078) | (1.084) | (1.067) | (1.332) | (1.295) | (0.350) | |  |
| Observations | | 344 | 343 | 343 | 344 | 344 | 344 | 344 | 344 | |  |
| Log Likelihood | | -441.248 | -445.785 | -443.578 | -441.254 | -444.556 | -444.401 | -449.244 | -448.954 | |  |
| Akaike Inf. Crit. | | 906.496 | 915.570 | 911.156 | 906.507 | 913.111 | 912.802 | 922.488 | 921.909 | |  |
| Note: | ^*^p<0.05; ^**^p<0.01; ^***^p<0.001 | | | | | | | | |  |  |

**Supplemental Table 1. Associations between immune function and child HAZ binned age.** REML regressions modeling interaction effects of immune cell count and age (binned by age group) on child height-for-age. Results are reported as beta coefficient (standard error). *Notes:* Cell counts are logged and z-scored; Mother’s height is z-scored; Residual WBC is fraction of WBC not included in cell count (e.g. for Mod 1, WBC = total WBC – T cell count), z-scored. ‘Respiratory’ is a binary variable (yes = 1) representing current respiratory illness, as diagnosed by a physician at time of visit.

| **Supplemental Table 2.** | | | | | | | | |
| --- | --- | --- | --- | --- | --- | --- | --- | --- |
|  | | | | | | | | |
|  | Dependent variable: Height (z-scored) | | | | | | | |
|  | Total T | Naïve | Non-naïve | CD4+ | CD8+ | B cells | NK cells | A:I ratio |
| Age (in years) | -0.009 | -0.008 | -0.012 | -0.009 | -0.012 | -0.011 | -0.007 | -0.001 |
|  | (0.022) | (0.022) | (0.022) | (0.022) | (0.022) | (0.022) | (0.021) | (0.022) |
| Sex (male = 1) | 0.032 | 0.035 | 0.033 | 0.037 | 0.030 | 0.029 | 0.044 | 0.047 |
|  | (0.094) | (0.094) | (0.094) | (0.094) | (0.094) | (0.094) | (0.094) | (0.094) |
| Mother’s height | 0.266^***^ | 0.267^***^ | 0.264^***^ | 0.263^***^ | 0.270^***^ | 0.269^***^ | 0.277^***^ | 0.276^***^ |
|  | (0.051) | (0.051) | (0.052) | (0.051) | (0.051) | (0.052) | (0.052) | (0.051) |
| Residual WBC | 0.045 | 0.046 | 0.055 | 0.049 | 0.052 | -0.007 | -0.032 | -0.059 |
|  | (0.118) | (0.118) | (0.119) | (0.118) | (0.119) | (0.131) | (0.132) | (0.135) |
| Weight-for-height | 0.051 | 0.051 | 0.050 | 0.053 | 0.049 | 0.050 | 0.058 | 0.061 |
|  | (0.054) | (0.054) | (0.054) | (0.053) | (0.054) | (0.054) | (0.054) | (0.054) |
| Current respiratory | -0.114 | -0.116 | -0.113 | -0.119 | -0.113 | -0.112 | -0.107 | -0.102 |
|  | (0.093) | (0.094) | (0.095) | (0.093) | (0.093) | (0.093) | (0.094) | (0.093) |
| Cell count : 3yrs and over: mother’s BMI | -0.111 | -0.050 | -0.046 | -0.077 | -0.055 | -0.068 | 0.038 | -0.053 |
|  | (0.133) | (0.063) | (0.045) | (0.117) | (0.118) | (0.111) | (0.091) | (0.064) |
| Cell count : under 3yrs : mother’s BMI | 0.311^t^ | 0.164 | 0.089 | 0.334^*^ | 0.198 | 0.053 | -0.216 | 0.191^*^ |
|  | (0.178) | (0.086) | (0.100) | (0.169) | (0.167) | (0.193) | (0.159) | (0.094) |
| Constant | -0.248 | -0.256 | -0.323 | -0.287 | -0.293 | 0.238 | 0.430 | 0.243 |
|  | (1.041) | (1.042) | (1.043) | (1.036) | (1.051) | (1.197) | (1.228) | (0.341) |
|  | | | | | | | | |
| Observations | 344 | 343 | 343 | 344 | 344 | 344 | 344 | 344 |
| Log Likelihood | -447.851 | -448.271 | -449.646 | -447.703 | -449.081 | -449.621 | -449.205 | -448.673 |
| Akaike Inf. Crit. | 917.702 | 918.542 | 921.291 | 917.407 | 920.162 | 921.242 | 920.411 | 919.346 |
| Note: | ^t^ p<0.10; ^*^p<0.05; ^**^p<0.01; ^***^p<0.001 | | | | | | | |

**Supplemental Table 2. Associations between immune function, mother's BMI, and child height-for-age.** REML regressions modeling three-way- interaction effects of immune cell count, age, and maternal energetic status (proxied by mother’s BMI, z-scored) on child height-for-age. Results are reported as beta coefficient (standard error). *Notes:* Cell counts are logged and z-scored; Mother’s height is z-scored; Residual WBC is fraction of WBC not included in cell count (e.g. for Mod 1, WBC = total WBC – T cell count), z-scored. ‘Respiratory’ is a binary variable (yes = 1) representing current respiratory illness, as diagnosed by a physician at time of visit.

| **Supplemental Table 3.** | | | | | | | | |
| --- | --- | --- | --- | --- | --- | --- | --- | --- |
|  | | | | | | | | |
|  | Dependent variable: Height (z-scored) | | | | | | | |
|  |  | | | | | | | |
|  | Total T | Naïve | Non-naïve | CD4+ | CD8+ | B cells | NK cells | A:I ratio |
|  | | | | | | | | |
| Cell count : 3yrs+ | -0.504^***^ | -0.205^**^ | -0.203^***^ | -0.478^***^ | -0.327^**^ | -0.339^**^ | -0.031 | -0.103 |
|  | (0.129) | (0.063) | (0.056) | (0.120) | (0.107) | (0.123) | (0.085) | (0.062) |
| Cell count : under 3yrs | -0.016 | 0.009 | -0.039 | 0.054 | -0.084 | 0.065 | -0.144 | 0.121 |
|  | (0.203) | (0.102) | (0.095) | (0.189) | (0.183) | (0.200) | (0.149) | (0.104) |
| Age (in years) | -0.047 | -0.040 | -0.026 | -0.045 | -0.037 | -0.031 | -0.004 | -0.007 |
|  | (0.025) | (0.026) | (0.022) | (0.025) | (0.024) | (0.026) | (0.023) | (0.027) |
| Child WHZ | 0.063 | 0.064 | 0.053 | 0.063 | 0.055 | 0.054 | 0.056 | 0.060 |
|  | (0.053) | (0.053) | (0.053) | (0.053) | (0.053) | (0.053) | (0.054) | (0.054) |
| Sex (male = 1) | 0.043 | 0.047 | 0.020 | 0.016 | 0.066 | 0.026 | 0.027 | 0.032 |
|  | (0.092) | (0.093) | (0.093) | (0.092) | (0.094) | (0.093) | (0.094) | (0.094) |
| Mother’s height | 0.284^***^ | 0.280^***^ | 0.280^***^ | 0.282^***^ | 0.280^***^ | 0.268^***^ | 0.271^***^ | 0.258^***^ |
|  | (0.051) | (0.052) | (0.051) | (0.051) | (0.051) | (0.051) | (0.052) | (0.052) |
| Residual WBC | 0.221 | 0.183 | 0.193 | 0.229 | 0.150 | 0.134 | 0.014 | -0.013 |
|  | (0.124) | (0.123) | (0.123) | (0.124) | (0.121) | (0.148) | (0.138) | (0.134) |
| Respiratory | -0.139 | -0.148 | -0.106 | -0.155 | -0.117 | -0.109 | -0.108 | -0.114 |
|  | (0.092) | (0.094) | (0.094) | (0.092) | (0.093) | (0.092) | (0.093) | (0.093) |
| Constant | -1.684 | -1.357 | -1.500 | -1.754 | -1.086 | -1.006 | 0.005 | 0.143 |
|  | (1.074) | (1.069) | (1.078) | (1.075) | (1.056) | (1.322) | (1.290) | (0.349) |
|  | | | | | | | | |
| Observations | 344 | 343 | 343 | 344 | 344 | 344 | 344 | 344 |
| Log Likelihood | -442.06 | -444.86 | -443.86 | -441.5 | -445.25 | -445.49 | -449.84 | -448.69 |
| R-squared (m) | 0.14 | 0.13 | 0.13 | 0.14 | 0.12 | 0.12 | 0.10 | 0.11 |
| R-squared (c) | 0.31 | 0.30 | 0.30 | 0.31 | 0.29 | 0.28 | 0.26 | 0.27 |
| Akaike Inf. Crit. | 906.12 | 911.72 | 909.73 | 905.47 | 912.50 | 912.99 | 921.67 | 919.39 |
| Note: | ^*^p<0.05; ^**^p<0.01; ^***^p<0.001 | | | | | | | |

**Supplemental Table 3. Associations between immune function, age, and child HAZ (adjust for weight-for-height, i.e. WHZ).** REML regressions modeling interaction effects of immune cell count and age, adjusting for potential direct effects of child energetic status (proxied by weight-for-height z-scores, i.e. WHZ) on child height-for-age. Notes: Cell counts are logged and z-scored; Mother’s height is z-scored; Residual WBC is fraction of WBC not included in cell count (e.g. for Mod 1, WBC = total WBC – T cell count), z-scored. ‘Respiratory’ is a binary variable (yes = 1) representing current respiratory illness, as diagnosed by a physician at time of visit. R-squared (m) is the marginal r-squared and represents the variance explained by the fixed effects; R-squared) is the conditional r-squared and represents the variance explained by both the fixed and random effects.

| **Supplemental Table 4.** | | | | | | | | |  |
| --- | --- | --- | --- | --- | --- | --- | --- | --- | --- |
|  | Dependent variable: Height (z-scored) | | | | | | | |  |
|  | Total T | Naïve | Non-naïve | CD4+ | CD8+ | B cells | NK cells | A:I ratio |  |
| Cell counts | -0.512^***^ | -0.196^**^ | -0.227^***^ | -0.435^***^ | -0.386^***^ | -0.349^**^ | -0.137 | -0.044 |  |
|  | (0.134) | (0.065) | (0.059) | (0.124) | (0.109) | (0.136) | (0.098) | (0.068) |  |
| Age (in years) | -0.040 | -0.032 | -0.010 | -0.035 | -0.028 | -0.025 | 0.024 | 0.001 |  |
|  | (0.030) | (0.030) | (0.027) | (0.030) | (0.029) | (0.030) | (0.028) | (0.031) |  |
| Sex (male=1) | 0.076 | 0.084 | 0.046 | 0.054 | 0.103 | 0.052 | 0.055 | 0.078 |  |
|  | (0.108) | (0.110) | (0.109) | (0.109) | (0.109) | (0.110) | (0.111) | (0.111) |  |
| Mother's height | 0.315^***^ | 0.310^***^ | 0.303^***^ | 0.311^***^ | 0.307^***^ | 0.287^***^ | 0.298^***^ | 0.288^***^ |  |
|  | (0.060) | (0.061) | (0.060) | (0.060) | (0.060) | (0.061) | (0.062) | (0.061) |  |
| Residual WBC | 0.211 | 0.147 | 0.209 | 0.190 | 0.143 | 0.122 | -0.041 | -0.132 |  |
|  | (0.148) | (0.148) | (0.148) | (0.149) | (0.144) | (0.174) | (0.166) | (0.160) |  |
| Respiratory | -0.118 | -0.140 | -0.091 | -0.141 | -0.103 | -0.094 | -0.112 | -0.131 |  |
|  | (0.108) | (0.111) | (0.111) | (0.108) | (0.109) | (0.110) | (0.111) | (0.111) |  |
| Helminthic (yes = 1) | -0.045 | -0.033 | -0.038 | -0.035 | -0.026 | -0.017 | -0.014 | 0.024 |  |
|  | (0.128) | (0.129) | (0.128) | (0.128) | (0.128) | (0.129) | (0.131) | (0.130) |  |
| Protozoal (yes = 1) | 0.078 | 0.091 | 0.089 | 0.091 | 0.088 | 0.133 | 0.109 | 0.145 |  |
|  | (0.130) | (0.132) | (0.130) | (0.131) | (0.131) | (0.131) | (0.134) | (0.133) |  |
| Constant | -1.625 | -1.083 | -1.736 | -1.453 | -1.090 | -0.947 | 0.359 | 0.373 |  |
|  | (1.276) | (1.271) | (1.286) | (1.280) | (1.248) | (1.546) | (1.532) | (0.398) |  |
|  | | | | | | | | |  |
| Observations | 246 | 245 | 245 | 246 | 246 | 246 | 246 | 246 |  |
| Log Likelihood | -315.18 | -317.75 | -315.10 | -316.44 | -316.44 | -318.79 | -321.32 | -322.33 |  |
| R-squared (m) | 0.16 | 0.14 | 0.16 | 0.15 | 0.15 | 0.13 | 0.12 | 0.11 |  |
| R-squared (c) | 0.37 | 0.35 | 0.38 | 0.35 | 0.37 | 0.36 | 0.36 | 0.34 |  |
| Akaike Inf. Crit. | 652.36 | 657.50 | 652.21 | 654.89 | 654.87 | 659.58 | 664.63 | 666.65 |  |
| **ANOVA table for random effect: Mother’s ID** | | | |  |  |  |  |  |  |
| Log Likelihood | -318.57 | -321.16 | -319.10 | -319.45 | -320.34 | -322.57 | -325.53 | -342.93 |  |
| Akaike Inf. Crit. | 657.14 | 662.33 | 658.20 | 658.90 | 660.68 | 665.15 | 671.07 | 703.86 |  |
| LRT^g^ | 6.79** | 6.83** | 7.99** | 6.01* | 7.81** | 7.57** | 8.43** | 6.57* |  |
| Note: | ^*^p<0.05; ^**^p<0.01; ^***^p<0.001 | | | | | | | | |

**Supplemental Table 4. Associations between immune function and child height-for-age adjusting for parasitic infection.** REML regressions modeling associations between immune cell count and child height-for-age, adjusting for current parasitic infection. Results are reported as beta coefficient (standard error). Notes: Cell counts are logged and z-scored; Mother’s height is z-scored; Residual WBC is fraction of WBC not included in cell count (e.g. for Mod 1, WBC = total WBC – T cell count), z-scored. ‘Respiratory’ is a binary variable (yes = 1) representing current respiratory illness, as diagnosed by a physician at time of visit. R-squared (m) is the marginal r-squared and represents the variance explained by the fixed effects; R-squared) is the conditional r-squared and represents the variance explained by both the fixed and random effects; LRT is the likelihood ratio test statistic.

| **Supplemental Table 5.** | | | | | | | | |
| --- | --- | --- | --- | --- | --- | --- | --- | --- |
|  | Dependent variable: Height (z-scored) | | | | | | | |
|  | Total T | Naïve | Non-naïve | CD4+ | CD8+ | B cells | NK cells | A:I ratio |
| Age (in years) | -0.037 | -0.029 | -0.006 | -0.031 | -0.026 | -0.018 | 0.022 | 0.002 |
|  | (0.030) | (0.031) | (0.027) | (0.030) | (0.029) | (0.031) | (0.029) | (0.032) |
| Sex (male = 1) | 0.076 | 0.083 | 0.052 | 0.050 | 0.106 | 0.054 | 0.056 | 0.078 |
|  | (0.108) | (0.110) | (0.108) | (0.108) | (0.109) | (0.110) | (0.111) | (0.112) |
| Mother’s height | 0.306^***^ | 0.303^***^ | 0.297^***^ | 0.300^***^ | 0.303^***^ | 0.284^***^ | 0.302^***^ | 0.287^***^ |
|  | (0.060) | (0.062) | (0.060) | (0.061) | (0.060) | (0.061) | (0.062) | (0.062) |
| Residual WBC | 0.236 | 0.161 | 0.234 | 0.224 | 0.150 | 0.115 | -0.047 | -0.130 |
|  | (0.150) | (0.149) | (0.148) | (0.150) | (0.145) | (0.174) | (0.166) | (0.161) |
| Respiratory | -0.127 | -0.146 | -0.108 | -0.155 | -0.106 | -0.103 | -0.115 | -0.131 |
|  | (0.108) | (0.112) | (0.110) | (0.109) | (0.109) | (0.111) | (0.112) | (0.111) |
| Helminthic (yes = 1) | -0.061 | -0.041 | -0.063 | -0.049 | -0.035 | -0.014 | -0.026 | 0.026 |
|  | (0.128) | (0.130) | (0.127) | (0.128) | (0.129) | (0.129) | (0.133) | (0.131) |
| Protozoal (yes = 1) | 0.064 | 0.086 | 0.047 | 0.087 | 0.076 | 0.134 | 0.101 | 0.146 |
|  | (0.131) | (0.132) | (0.131) | (0.130) | (0.132) | (0.131) | (0.135) | (0.134) |
| Cell count : 3yrs and over | -0.589^***^ | -0.216^**^ | -0.287^***^ | -0.525^***^ | -0.418^***^ | -0.398^**^ | -0.165 | -0.048 |
|  | (0.148) | (0.071) | (0.066) | (0.138) | (0.121) | (0.142) | (0.108) | (0.075) |
| Cell count : under 3yrs | -0.245 | -0.111 | -0.048 | -0.105 | -0.270 | -0.084 | -0.031 | -0.028 |
|  | (0.260) | (0.134) | (0.109) | (0.249) | (0.219) | (0.276) | (0.192) | (0.137) |
| Constant | -1.869 | -1.222 | -1.964 | -1.781 | -1.158 | -0.926 | 0.441 | 0.359 |
|  | (1.291) | (1.287) | (1.283) | (1.293) | (1.254) | (1.545) | (1.539) | (0.411) |
|  | | | | | | | | |
| Observations | 246 | 245 | 245 | 246 | 246 | 246 | 246 | 246 |
| Log Likelihood | -314.80 | -318.50 | -314.39 | -315.68 | -316.75 | -318.52 | -321.76 | -323.29 |
| R-squared (m) | 0.16 | 0.14 | 0.17 | 0.16 | 0.15 | 0.14 | 0.12 | 0.11 |
| R-squared (c) | 0.38 | 0.36 | 0.39 | 0.38 | 0.37 | 0.36 | 0.36 | 0.31 |
| Akaike Inf. Crit. | 653.59 | 661.00 | 652.78 | 655.35 | 657.51 | 661.04 | 667.51 | 670.58 |
| Note: | ^*^p<0.05; ^**^p<0.01; ^***^p<0.001 | | | | | | | |

**Supplemental Table 5. Associations between immune function and child height-for-age by age group, adjusting for parasitic infection.** REML regressions modeling interaction effects of immune cell count and age on child height-for-age, adjusting for current parasitic infection. Results are reported as beta coefficient (standard error). Notes: Cell counts are logged and z-scored; Mother’s height is z-scored; Residual WBC is fraction of WBC not included in cell count (e.g. for Mod 1, WBC = total WBC – T cell count), z-scored. ‘Respiratory’ is a binary variable (yes = 1) representing current respiratory illness, as diagnosed by a physician at time of visit. R-squared (m) is the marginal r-squared and represents the variance explained by the fixed effects; R-squared) is the conditional r-squared and represents the variance explained by both the fixed and random effects; LRT is the likelihood ratio test statistic.

| Supplemental Table 6. F-statistics (ANOVA (type II)) testing differences by parasitic infection status | | | | | | | | |  |
| --- | --- | --- | --- | --- | --- | --- | --- | --- | --- |
|  | Total T | Naïve | Non-naïve | CD4+ | CD8+ | B cells | NK cells | A:I ratio | WHZ |
| Age (years) | 29.96*** | 36.06*** | 4.03* | 27.54*** | 20.21*** | 26.91*** | 15.64** | 78.10*** | 0.41 |
| Helminthic | 0.01 | 0.14 | 0.54 | 0.07 | 0.06 | 0.24 | 1.66 | 0.84 | 0.35 |
| Age (years) | 33.22*** | 41.40*** | 3.32* | 31.08*** | 21.78*** | 27.39*** | 12.71** | 78.78*** | 0.4 |
| Protozoal | 0.12 | 0.07 | 0.17 | 0.35 | 0.01 | 0.52 | 3.09 | 2.81 | 3.04^t^ |
|  |  |  |  |  |  |  |  |  |  |

Supplemental Figure 1. Mixed effects linear model shows that for infants (under 3yrs old) whose mothers have above the mean BMI, there is a positive association between T-cell count and height-for-age, whereas for older children (over 3yrs old), there remains a negative association between cell count and height-for-age, regardless of maternal BMI. Model adjusts for age, sex, mother’s height, current respiratory infection and residual WBC, and have a random effect for mother’s ID.

Supplemental Figure 2. Adjusting for age, sex, mother’s height, current respiratory infection and residual WBC, children that are below the mean for weight-for-height (WHZ) are overall shorter than above the mean WHZ children. Also, the negative association between height-for-age and T-cell count exists only for the above the mean WHZ group.
